# Coefficient of glucose variation is independently associated with mortality in critically ill patients receiving intravenous insulin

**DOI:** 10.1186/cc13851

**Published:** 2014-04-30

**Authors:** Michael J Lanspa, Justin Dickerson, Alan H Morris, James F Orme, John Holmen, Eliotte L Hirshberg

**Affiliations:** 1 Division of Pulmonary and Critical Care Medicine, University of Utah School of Medicine, Salt Lake City, UT, USA; 2 Division of Pulmonary and Critical Care Medicine, Intermountain Medical Center, Murray, UT, USA; 3 Homer Warner Center, Intermountain Healthcare, Salt Lake City, UT, USA; 4 Division of Pediatric Critical Care, University of Utah School of Medicine, Salt Lake City, UT, USA

## Abstract

**Introduction:**

Both patient- and context-specific factors may explain the conflicting evidence regarding glucose control in critically ill patients. Blood glucose variability appears to correlate with mortality, but this variability may be an indicator of disease severity, rather than an independent predictor of mortality. We assessed blood glucose coefficient of variation as an independent predictor of mortality in the critically ill.

**Methods:**

We used eProtocol-Insulin, an electronic protocol for managing intravenous insulin with explicit rules, high clinician compliance, and reproducibility. We studied critically ill patients from eight hospitals, excluding patients with diabetic ketoacidosis and patients supported with eProtocol-insulin for < 24 hours or with < 10 glucose measurements. Our primary clinical outcome was 30-day all-cause mortality. We performed multivariable logistic regression, with covariates of age, gender, glucose coefficient of variation (standard deviation/mean), Charlson comorbidity score, acute physiology score, presence of diabetes, and occurrence of hypoglycemia < 60 mg/dL.

**Results:**

We studied 6101 critically ill adults. Coefficient of variation was independently associated with 30-day mortality (odds ratio 1.23 for every 10% increase, *P* < 0.001), even after adjustment for hypoglycemia, age, disease severity, and comorbidities. The association was higher in non-diabetics (OR = 1.37, *P* < 0.001) than in diabetics (OR 1.15, *P* = 0.001).

**Conclusions:**

Blood glucose variability is associated with mortality and is independent of hypoglycemia, disease severity, and comorbidities. Future studies should evaluate blood glucose variability.

## Introduction

The optimal management of blood glucose in the ICU remains unclear. A study of surgical ICU patients in Leuven, Belgium, demonstrated that insulin therapy aimed at achieving blood glucose between 80 and 110 mg/dL (tight glucose control) decreased subject mortality compared to conventional treatment (maintenance of blood glucose between 180 and 200 mg/dL) [1]. Subsequent studies evaluating the role of insulin therapy in the ICU either failed to confirm these results, or were terminated early due to high hypoglycemia rates [2-6]. The largest prospective multicenter trial to date (Normoglycaemia in Intensive Care Evaluation and Survival Using Glucose Algorithm Regulation, NICE-SUGAR) reported an increase in 90-day mortality for the group with an 80 to 110 mg/dL blood glucose target when compared to a target of <180 mg/dL [7]. Despite continued uncertainty in glucose management, professional societies currently recommend moderate glucose control for all critically ill adult patients [8,9].

One explanation for the incongruencies between studies is that mean blood glucose (reported most often in relation to blood glucose target) may not be the most important aspect of glucose control in critically ill patients. Some authors postulate that other glucose metrics, including within-patient glycemic variability, may be as important, or more important than a mean blood glucose target [10,11]. Multiple studies have demonstrated an association between glycemic variability and mortality [12-19]. Several measures of glycemic variability have been studied: SD, coefficient of variation, glycemic lability index, and mean amplitude of glycemic excursion [20]. Coefficient of variation (SD/mean × 100%) normalizes glycemic variability at different mean blood glucose values. Coefficient of variation correlates with mortality in the ICU [13,15,17-19].

The association of glycemic variability with mortality could be independent of critical illness, a covariate, or both. Glycemic variability may be a unique patient attribute, it may be the result of unnecessary inter-physician variation while attempting to control blood sugar with insulin, or it may be a consequence of hypoglycemia, believed to confer harm. We studied the association between coefficient of variation of glucose and mortality. While this association is well-documented in previous studies [12-19], in this study, we eliminated inter-physician variation by standardizing physician decisions with eProtocol-Insulin, an explicit, replicable, electronic protocol for managing blood glucose in ICU patients [21,22]. Clinician compliance with eProtocol-insulin recommendations is 95%, and the implementation of eProtocol-insulin has resulted in clinical reproducibility of blood glucose metrics across multiple environments [21-23]. We also assessed whether the association between glycemic variability and mortality was independent of hypoglycemia and other patient attributes.

## Methods

### Data collection

**Figure 1:**
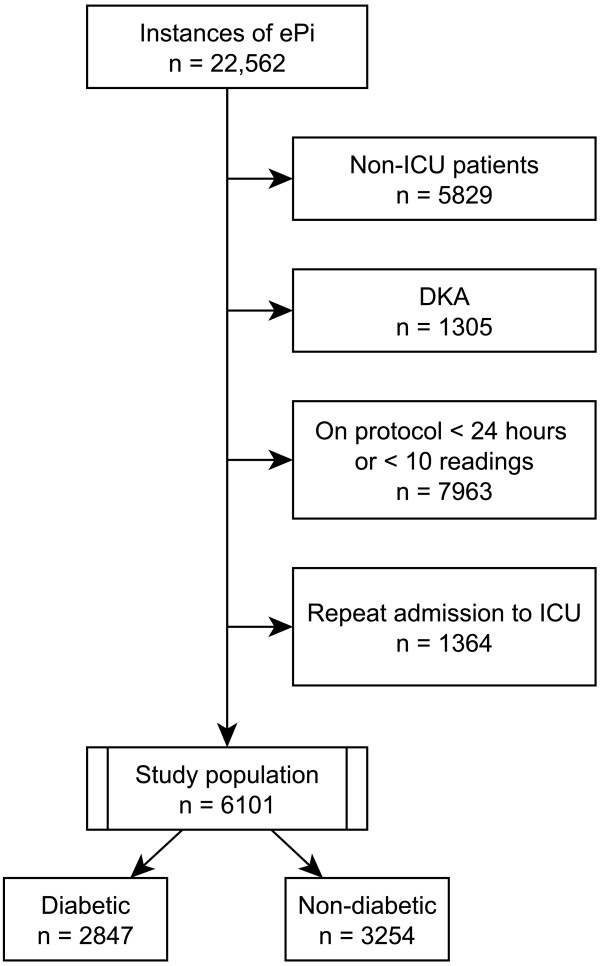
**Study population and excluded patients.** DKA, diabetic ketoacidosis; ePi, eProtocol-insulin.

Using Intermountain Healthcare’s electronic medical record, we performed a retrospective cohort analysis of all patients supported with eProtocol-insulin from November 2006 to August 2012 (Intermountain Healthcare Institutional Review Board, number 1008548, approved this study, and allowed waiver of informed consent due to its retrospective nature). Patients were drawn from 14 different ICUs from 8 different hospitals. These open ICUs included medical (2), surgical (2), and mixed (9) patient populations, teaching (2) and non-teaching (12) ICU’s. Diagnostic categories of reason for admission were not assessed, although we excluded patients with diabetic ketoacidosis, as we believe they comprised a different patient experience than the typical ICU patient on intravenous insulin for blood glucose management. Similarly, we also excluded patients supported with eProtocol-insulin for <24 hours or with <10 blood glucose measurements. We included only a patient’s first ICU admission during the study period. We stratified the data by presence or absence of diabetes mellitus (Figure 1), determined by the International Statistical Classification of Diseases and Related Health Problems, 9^th^ revision: (ICD-9) 249.x-250.x code. We calculated the acute physiology component of the acute physiology and chronic health evaluation (APACHE)-II score (excluding age and chronic comorbidities) to avoid co-linearity in our regression model. We calculated the Charlson comorbidity index using ICD-9 codes [24,25].

### Glucose management

The eProtocol-Insulin algorithm is as follows:

$$\begin{array}{l} \text{Initial}\;\text{infusion}\;\text{rate}=0.0126\times \text{measured}\;\text{glucose}\left(\mathrm{mg}/\mathrm{dL}\right)\\ \times \text{weight}÷70\mathrm{kg}\\ \text{New}\;\text{infusion}\;\text{rate}=\text{old}\;\text{rate}\\ +\frac{\sqrt{\text{old}\;\text{rate}\times \left(\text{observed}\;\mathrm{dg}/\mathrm{dt}-\text{desired}\;\mathrm{dg}/\mathrm{dt}\right)\times \text{glucose}-\text{target}}}{\text{desired}\;\mathrm{dg}/\mathrm{dt}} \end{array}$$

Blood glucose management in Intermountain Healthcare ICUs includes standardized institutional processes. All ICUs used the OneTouch SureStep (LifeScan, Milpitas, CA, USA) bedside glucose meter until 2010, when all facilities switched to the HemoCue (Quest Diagnostics, Cypress CA, USA) glucose meter. All glucose meters were calibrated nightly. All ICUs administered intravenous (IV) regular insulin using a smart pump in plastic tubing with a concentration of 1 unit/mL. The time interval of blood glucose measurements was explicitly determined by eProtocol-insulin, based on glucose stability. All study ICUs have a 2:1 patient/nurse ratio. eProtocol-insulin does not contain detailed nutrition rules but adjusts recommendations based on glucose-calories above or below half the estimated basal caloric requirement [26]. Both the decision to initiate eProtocol-insulin and the blood glucose target were determined by the individual practitioner at a target of either 95 mg/dL (corresponding to an expected range of 80 to 110 mg/dL), or at 115 mg/dL (corresponding to an expected range of 90 to 140 md/dL). Shortly after the publication of NICE-SUGAR, most physicians switched from the 95 mg/dL to the 115 mg/dL target [27]. eProtocol-insulin discontinues IV insulin and recommends concentrated IV dextrose when blood glucose is <60 mg/dL. eProtocol-insulin support is discontinued if patients are receiving bolus feeds.

### Statistical analysis

Given the non-parametric distribution of the data, we compared central tendencies between groups using the Mann–Whitney *U*-test, and compared proportions using the Chi-squared test. We performed multivariable logistic regression to assess the impact of blood glucose coefficient of variation on 30-day mortality in the diabetic and non-diabetic populations. In the regression models, we adjusted for age, acute physiology score, Charlson comorbidity score, presence of diabetes, glucose coefficient of variation and occurrence of hypoglycemia (at least one blood glucose <60 mg/dL). We stratified the models for presence or absence of diabetes. Because we anticipated the coefficient of variation would vary co-linearly with hypoglycemia, we decided a priori to use a likelihood ratio test to determine the independent contribution of coefficient of variation on mortality in a model that included hypoglycemia. In all multivariable regression models, we assessed the role of multi-co-linearity with variance inflation factor, the existence of specification error with a link test, and the calibration of the model with Hosmer-Lemeshow goodness-of-fit test. All displayed *P*-values are two-sided. We analyzed the data with Stata-12 statistical software (StataCorp LP, College Station, TX, USA).

## Results

We identified 6,101 patients, of whom 46.7% had diabetes. Diabetic patients were older, had higher mean blood glucoses, higher SD of glucose, and higher coefficients of variation than non-diabetic patients (Table 1). Diabetic patients had greater comorbidity scores (6 versus 2) and lower acute physiology scores (19 versus 21) than non-diabetic patients. Diabetic patients were also more likely than non-diabetic patients to have hypoglycemia. The overall rate of hypoglycemia in all patients (glucose <60 mg/dL) and severe hypoglycemia (<40 mg/dL) were 24% and 3%. There was no difference in 30-day mortality between diabetic and non-diabetic patients (*P* = 0.15).

**Table 1:** Patient characteristics

	**All patients n = 6101**	**Diabetic patients n = 2847**	**Non-diabetic patients n = 3254**	***P*-value**
Age (years)	65 (53 to 75)	67 (58 to 76)	63 (49 to 74)	<0.0001
Female (%)	40.5	41.4	39.8	0.209
Mean blood glucose (mg/dL)	123 (112 to 135)	129 (117 to 143)	119 (109 to 129)	<0.0001
Glucose (SD)	32 (24 to 44)	38 (29 to 53)	28 (22 to 37)	<0.0001
Coefficient of variation (%)	27 (21 to 34)	30 (24 to 39)	24 (19 to 30)	<0.0001
Charlson comorbidity score	4 (2 to 7)	6 (4 to 9)	2 (1 to 4)	<0.0001
Acute physiology score	20 (14 to 26)	19 (13 to 25)	21 (15 to 27)	<0.0001
Hypoglycemia (%)	24.0	25.5	22.6	0.009
30-day mortality (%)	20.3	20.1	20.5	0.1551

Continuous data are listed as medians with interquartile ranges in parentheses. The *P*-value is a comparison between diabetic and non-diabetic patients. Hypoglycemia is defined as having at least one measured blood glucose <60 mg/dL while supported with eProtocol-insulin. The acute physiology score was calculated at the time of ICU admission.

**Figure 2:**
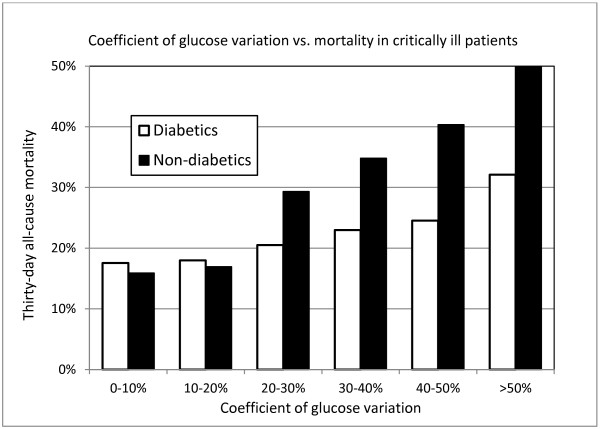
**Thirty-day mortality as a function of blood glucose coefficient of variation, stratified for presence of diabetes.** The associations were statistically significant in both groups (*P* <0.001).

Univariable analysis of the unadjusted coefficient of variation was strongly associated with mortality for the entire cohort (odds ratio (OR) 1.25 for every 10% increase, 95% CI = 1.19, 1.32, *P* <0.001). Unadjusted coefficient of variation was more strongly associated with mortality in non-diabetics (OR 1.48, 95% CI = 1.37, 1.62, *P* <0.001) than in diabetics (OR 1.14, 95% CI = 1.06, 1.23, *P* <0.001, Figure 2).

After multivariable regression model adjustment for age, gender, disease severity, comorbidities, diabetic status, blood glucose target, and hypoglycemia, the coefficient of variation was still associated with mortality (OR 1.23 for every 10% increase, 95% CI = 1.16, 1.31, *P* <0.001, Table 2). This association was significantly greater in non-diabetic patients (OR 1.37, 95% CI = 1.25, 1.50, *P* <0.001) than diabetic patients (OR 1.15, 95% CI = 1.06, 1.24, *P* = 0.001). The effect of coefficient of variation on mortality was independent of hypoglycemia in the entire cohort (*χ*_2_ = 45.62, *P* <0.001), in diabetic patients (*χ*_2_ = 11.20, *P* = 0.001), and in non-diabetic patients (*χ*_2_ = 44.40, *P* <0.001). Other covariates associated with increased mortality were age, acute physiology score, Charlson comorbidity index, and occurrence of blood glucose <60 mg/dL. The presence of diabetes was associated with decreased mortality in the multivariate model (OR 0.64, *P* <0.001). The association between coefficient of variation and mortality was maintained in the subset of patients who never had a hypoglycemic event (OR 1.20, 95% CI = 1.12, 1.29, *P* <0.001).

**Table 2:** Logistic regressions of 30-day all-cause mortality, using covariates of age, acute physiology score, and Charlson score

	**Odds ratio (95% CI) (model 1)**	***P*-value (model 1)**	**Odds ratio (95% CI) (model 2)**	***P*-value (model 2)**
**All patients (n = 6,101)**				
Age (decade)	1.16 (1.10, 1.22)	<0.001	1.15 (1.09, 1.22)	<0.001
Charlson score	1.21 (1.14, 1.28)	<0.001	1.20 (1.13, 1.27)	<0.001
Acute physiology score	1.09 (1.07, 1.10)	<0.001	1.08 (1.07, 1.10)	<0.001
Hypoglycemia	1.53 (1.33, 1.77)	<0.001	1.34 (1.16, 1.56)	<0.001
Diabetes	0.63 (0.54, 0.74)	<0.001	0.55 (0.46, 0.64)	<0.001
Interaction of age and diabetes	1.09 (0.99, 1.20)	0.070	1.11 (1.01, 1.22)	0.033
Interaction of Charlson and acute physiology scores	1.00 (0.99, 1.00)	0.005	1.00 (0.99, 1.00)	0.016
Coefficient of variation (10%)			1.23 (1.16, 1.31)	<0.001
**Diabetic patients (n = 2,847)**				
Age (decade)	1.29 (1.19, 1.40)	<0.001	1.30 (1.19, 1.41)	<0.001
Charlson score	1.25 (1.14, 1.37)	<0.001	1.25 (1.14, 1.36)	<0.001
Acute physiology score	1.12 (1.09, 1.16)	<0.001	1.12 (1.08, 1.16)	<0.001
Hypoglycemia	1.42 (1.15, 1.75)	<0.001	1.30 (1.05, 1.61)	0.020
Interaction of Charlson and acute physiology scores	0.99 (0.99, 1.00)	0.001	0.99 (0.99, 1.00)	0.018
Coefficient of variation (10%)			1.15 (1.06, 1.24)	0.001
**Non-diabetic patients (n = 3,254)**				
Age (decade)	1.14 (1.08, 1.21)	<0.001	1.13 (1.07, 1.20)	<0.001
Charlson score	1.31 (1.20, 1.43)	<0.001	1.30 (1.18, 1.42)	<0.001
Acute physiology score	1.09 (1.07, 1.11)	<0.001	1.08 (1.06, 1.10)	<0.001
Hypoglycemia	1.65 (1.36, 2.01)	<0.001	1.35 (1.10, 1.66)	0.004
Interaction of Charlson and acute physiology scores	0.99 (0.99, 1.00)	0.001	0.99 (0.99, 1.00)	0.003
Coefficient of variation (10%)			1.37 (1.25, 1.50)	<0.001

Model 2 includes coefficient of variation. Age is mean-centered. Both age and coefficient of variation were scaled for clinical relevance. Acute physiology score is calculated from the acute physiology and chronic health evaluation II (APACHE II), omitting age and chronic health parameters. Hypoglycemia is defined as having at least one measured blood glucose <60 mg/dL while supported with eProtocol-insulin. Interactions were included to account for multi-co-linearity. Initial model also included gender and choice of blood glucose target, both of which were omitted from the final model due to non significance. The likelihood ratio test demonstrates an independent effect of coefficient of variation on mortality in the diabetic (*χ*_2_ = 11.20, *P* = 0.001), non-diabetic (*χ*_2_ = 44.40, *P* <0.001), and combined groups (*χ*_2_ = 45.62 *P* <0.001).

## Discussion

We demonstrate that coefficient of blood glucose variation is independently associated with 30-day mortality in the critically ill population. This association is independent of age, disease severity, comorbidities, diabetic status, and hypoglycemia. While previous work in this area has demonstrated an association of coefficient of variation and mortality, this study adds value to the literature in three ways: (1) we employed an explicit electronic protocol to significantly reduce inter-clinician variability in the method of insulin titration; (2) we demonstrated that coefficient of variation is associated with mortality even in diabetic patients, and (3) we accounted for hypoglycemia when analyzing coefficient of variation. Notably, our rates of severe hypoglycemia (<40 mg/dL) were significantly lower than reported in the lower glucose target of the NICE-SUGAR study (3.4% versus 6.8%, *P* <0.0001) [28].

Previous studies investigating the effects of glucose variability either lacked any specific protocol for insulin management [12,15], or allowed variation with clinician judgment in adjustment of insulin and in frequency of blood glucose measurements [1,4,7]. Several previous studies have either omitted diabetic status from analysis, or did not stratify the analysis based on diabetic status [12,14-16,29]. Both Krinsley and Sechterberger stratified analysis on diabetic status, and demonstrated that coefficient of variation was associated with mortality in non-diabetic, but not in diabetic patients [17-19]. We believe it necessary to stratify analysis upon diabetic status. Diabetic patients behave differently than non-diabetic patients regarding intravenous insulin therapy [16-19,30-33]. Although prior studies also demonstrated that hypoglycemia and glycemic variability contribute to mortality, many do not demonstrate that the association of glycemic variability to mortality is independent of hypoglycemia [12,14,16,29,34]. From these studies we know neither how much collinearity exists between hypoglycemia and the chosen metric of glycemic variability, nor how much of the association between glycemic variability and mortality is driven by hypoglycemia. Krinsley demonstrated that the association still holds even after excluding severe hypoglycemia, suggesting that the relationship is not driven by hypoglycemia [13]. Our data are congruent with Krinsley’s finding that the association of coefficient of variation on mortality is maintained in patients without hypoglycemia. Furthermore, our data demonstrate that the association of coefficient of variation and mortality is independent of hypoglycemia even when hypoglycemic patients are included in the model. To our knowledge, we are the first to demonstrate that the association between blood glucose coefficient of variation and mortality occurs in diabetic patients, and the association occurs when using data from a replicable clinician decision method (eProtocol-insulin).

Blood glucose variability seems to be an important characteristic of a patient’s glucose homeostasis, but also may be a characteristic of the clinician’s treatment. Less glycemic variability may reflect more precise glucose management with IV insulin, and may result from more fastidious medical care, leading to improved outcomes [10]. Confounding from clinician variation in medical care is likely in studies that lack an explicit and reproducible method for insulin titration and blood glucose management. Such confounding is unlikely in this study as a unique feature of this study is its reliance on eProtocol-insulin, an electronic protocol that uses explicit and detailed rules for intravenous insulin and blood glucose measurement to achieve 95% clinician compliance and clinical reproducibility [21-23]. Hence, there is little inter-clinician variability in glucose management in the current study population.

Glycemic variability may cause harmful effects. Among patients with type II diabetes, increased glycemic variability is associated with increased protein kinase C-β, a marker of oxidative stress [35]. Increased glycemic variability also increases oxidative stress at the cellular level [36,37]. Nevertheless, no study, including this one, has demonstrated a causal relationship between glycemic variability and death in the ICU.

Glycemic variability may simply be an epiphenomenon of a critically ill patient’s inability to maintain homeostasis. While this study cannot determine the validity of this possibility, some of its results offer insight into the relationship between glycemic variability and mortality. Because the eProtocol-insulin was identical for diabetic and non-diabetic patients, the differences in glycemic variability between these two groups are likely caused by patient-specific factors. A non-diabetic patient who requires IV insulin has, by definition, already lost the ability to maintain glucose homeostasis. At least some component of this glucose dysregulation is reasonably inferred to be the result of critical illness, not its cause. We found the association between blood glucose coefficient of variation and mortality was significantly greater in non-diabetic than in diabetic patients. Although we adjusted for disease severity, the acute physiology score we used is far from a comprehensive assessment of disease severity. We are unable to account for every possible indicator of disease severity, and it is possible that unmeasured indicators of disease severity confound the association between glycemic variability and mortality. If the increased mortality was caused solely by increased glycemic variability, we would expect similar associations in diabetic and non-diabetic patients, and expect that diabetic patients (with greater glycemic variability), would have greater mortality. Our results support neither of these expectations. While we think it is likely that some component of glycemic variability is caused by disease severity, we recognize that increased glycemic variability itself may have an adverse effect on patient outcome. Our data do not allow for inferences of mortality benefit from reduction of glycemic variability.

Our study used a threshold of 60 mg/dL for hypoglycemia. This threshold is lower than the 70 mg/dL threshold commonly reported in the literature. Our rationale for this threshold is that the protocol becomes discontinuous at <60 mg/dL (insulin is suspended and glucose is administered), which may have significant effects on glycemic variation.

While there was no statistically significant difference in mortality rate among diabetic and non-diabetic patients, diabetes was associated with reduced mortality in the multivariable analysis. The association of diabetes with comparable or decreased mortality in the critically ill is well-documented in the literature [18,19,31,38-42]. One explanation is that acute dysglycemia may confer less harm in the diabetic patient who has developed a tolerance to the complications of hyperglycemia. The GLUT4 transporter, a signaling molecule that affects myocardial function, is downregulated with chronic hyperglycemia, and is upregulated with administration of insulin [43,44]. Another possible explanation is selection bias. The high proportion of study patients with diabetes suggests that physicians were more likely to use eProtocol-insulin in diabetic than non-diabetic patients. Non-diabetic patients had greater severity of illness than diabetic patients (by acute physiology score). We suspect the severely ill non-diabetic patients were more likely to be selected for blood glucose management with eProtocol-insulin, and therefore diabetes (in less severely ill patients) was associated with reduced mortality.

This study’s results are limited, although we studied many patients from a heterogenous population (community and referral hospitals, medical and surgical ICUs, private and academic hospitals). Generalizability of the results is limited by our use of eProtocol-insulin and by the retrospective analysis. Physicians were not required to use eProtocol-insulin, and we do not know how many patients were managed without eProtocol-insulin. We suspect selection bias because patients supported with eProtocol-insulin may be substantially different than those who were not. For example, the proportion of diabetic patients (47%) was much higher in this study than expected for a typical ICU. The ICD-9 determination of diabetes did not require hemoglobin A1c values. Undiagnosed diabetes might then be erroneously categorized as non-diabetic. We do not have the data to pursue further the selection bias issue. We excluded a large number of patients, including those with diabetic ketoacidosis or who were supported with eProtocol-insulin for <1 day. While patients on this study did not receive bolus feeding, we did not quantify enteral or parenteral glucose amount, duration, or frequency. We expect such factors are associated with glucose variability, and should be controlled in future prospective studies. Blood glucose measurements from capillary glucose meters have known analytic inaccuracies [45,46], although the meters were calibrated daily according to industry standards.

The clinically relevant question is whether reduction of glycemic variability will improve outcomes. The answer to that question will require a prospective study aimed at reducing glycemic variability. The large prospective studies looking at glucose management in the critically ill have compared different mean blood glucose targets, with little or less attention paid to other glucose metrics, such as glycemic variability [1,4,6,7]. Furthermore the relationship between glycemic variability, blood glucose target range, and the method of blood glucose control is largely ignored in previously published prospective studies. Multiple studies have reported incongruent results. We believe these disparate results follow from testing different protocols in different populations, with varying, and usually unreported, clinician compliance. Consequently, we understand little more about best practice for blood glucose management than we did before the publication by van den Berghe *et al*. [1]. Future studies should employ replicable protocols, and should include assessment and treatment of glycemic variability, among other glucose metrics. Constructing such a study may be difficult in human subjects without deliberately inducing undesirable variations in blood glucose.

## Conclusion

In critically ill patients treated with an explicit, electronic insulin protocol (eProtocol-insulin), blood glucose coefficient of variation was associated with 30-day mortality. This association was present in diabetic as well as in non-diabetic patients. The association was independent of hypoglycemia, blood glucose target, age, disease severity, and comorbidities. Future studies should include assessment of blood glucose variability.

## Key messages

• Blood glucose coefficient of variation is associated with 30-day mortality in ICU patients receiving intravenous insulin.

• This association also persists in diabetic patients, and is independent of hypoglycemia.

• This association is unlikely to be the result of inter-physician variation, as we standardized physician decisions with an explicit, replicable insulin protocol.

## Abbreviations

ICD-9: International Statistical Classification of Diseases and Related Health Problems 9^th^ revision; IV: intravenous; NICE-SUGAR: Normoglycaemia in intensive care evaluation and survival using glucose algorithm regulation; OR: odds ratio.

## Competing interests

The authors declare that they have no competing interests.

## Authors’ contributions

ML contributed to study conception and design, data analysis, statistical analysis, and drafting the manuscript. EH contributed to study conception and design, and revision of the manuscript for important intellectual content. JD contributed to study design, statistical analysis, and revision of the manuscript for important intellectual content. JH contributed to data analysis, revision of manuscript for important intellectual content. JO and AM contributed to study conception and design, and revision of the manuscript for important intellectual content. All authors read and approved the final manuscript. ML takes responsibility for the integrity of the work as a whole, from inception to the published article.
